# Increase of central foveal and temporal choroidal thickness in patients with inactive thyroid eye disease

**DOI:** 10.1186/s12886-021-01804-x

**Published:** 2021-01-12

**Authors:** Joohyun Kim, Sumin Yoon, Sehyun Baek

**Affiliations:** Department of Ophthalmology, Korea University College of Medicine, 148 Gurodong-ro, Guro-gu, 08308 Seoul, Korea

**Keywords:** Choroidal thickness, Optical coherence tomography, Thyroid eye disease

## Abstract

**Background:**

In this study, we aimed to compare the choroidal thickness between a group of Korean patients with inactive thyroid eye disease (TED) and a control group of Korean patients and to analyze the variables affecting choroidal thickness.

**Methods:**

Patients diagnosed with inactive TED and without TED who underwent optical coherence tomography and axial length measurements were included and classified into the TED group and control group. Choroidal thickness was measured using images acquired in enhanced depth imaging (EDI) mode by cirrus HD-OCT (Carl Zeiss Meditec Inc., Dublin, CA, UAS) at the central fovea and points 1.5 mm nasal and 1.5 mm temporal from the central fovea using a caliper tool provided by OCT software.

**Results:**

The mean central foveal choroidal thickness was 294.2 ± 71.4 µm and 261.1 ± 47.4 µm in the TED and control groups, respectively, while the mean temporal choroidal thickness was 267.6 ± 67.5 µm and 235.7 ± 41.3 µm in the TED and control groups, respectively, showing significant differences between the two groups (*P* = 0.011, *P* = 0.008). The mean nasal choroidal thickness was 232.1 ± 71.7 µm and 221.1 ± 59.9 µm in the TED and control groups, respectively, showing no significant difference between the two groups (*P* = 0.421). Multivariate regression analysis showed the factors affecting central foveal choroidal thickness were age, axial length, and degree of exophthalmos, and factors affecting temporal choroidal thickness were age and degree of exophthalmos.

**Conclusions:**

Central foveal and temporal choroidal thickness were significantly thicker in patients with inactive TED than in control subjects, while age, axial length, and degree of exophthalmos were identified as major factors affecting choroidal thickness.

## Background

Thyroid eye disease (TED) is an autoimmune disorder characterized by proliferation of the orbital fat tissue and inflammation of the orbital connective tissue and extraocular muscles. TED is observed in approximately 25–50% of patients with Graves’ disease and 2% of patients with thyroiditis [1]. Although most patients with TED present with mild symptoms, the condition may progress into a more severe form in approximately 3–5% of the patients. TED involves proliferation of orbital fibro-adipose tissues and infiltration of inflammatory cells, causing an increase in orbital tissue volume. Clinical signs of TED include dry eyes, exposure keratopathy, exophthalmos, diplopia, and decreased visual acuity [2].

The choroid supplies 70% or more of the total blood volume involved in ocular circulation, and provides oxygen and nutrients to the outer retinal layer. It has been reported that central serous chorioretinopathy, age-related macular degeneration, ocular inflammatory disease, diabetes mellitus, and glaucoma may affect choroidal circulation, leading to changes in choroidal thickness [3–9].

Recently, there have been a few studies on changes in choroidal thickness in patients with TED and it has been reported that patients with higher clinical activity scores (CAS) tend to show increased choroidal thickness [10, 11]. However, the exact cause of choroidal thickening has not yet been identified, and there is no clinical study that included only patients with TED who have low CAS. Accordingly, the authors of the present study aimed to compare the choroidal thickness between patients with inactive TED (TED group) and control group and analyze the variables affecting choroidal thickness to understand the pathophysiology of changes in choroidal thickness in patients with TED.

## Methods

Among 58 patients who visited the Department of Ophthalmology in Korea University Guro Hospital between March 2017 and June 2018 and were subsequently diagnosed with TED, the medical records of 49 patients (15 males and 34 females) with inactive TED and CAS < 3 were analyzed retrospectively. For the control group, we included 49 consecutive patients (15 male and 34 female) without TED who underwent optical coherence tomography and axial length measurements since March 2017 in the Department of Ophthalmology. The patients provided informed consent. The study was conducted following the tenets of the Helsinki Declaration and was approved by the Institutional Review Board of Korea University Medical Center.

Diagnosis of TED was defined as meeting two or more of the following criteria: (1) abnormal findings in thyroid function tests, (2) having degree of exophthalmos ≥ 17 mm or difference of ≥ 2 mm between the two eyes as measured using an exophthalmometer; presenting with other symptoms, including lid retraction or lid lag, and (3) enlarged extraocular muscles confirmed by orbital computed tomography. Patients diagnosed with glaucoma, pachychroid disease spectrum, choroidal disease, or retinopathy; patients with refractive power < -6 or ≥ + 6 diopter; and patients who underwent previous refractive surgery were excluded.

The patient characteristics investigated included age, gender, intraocular pressure, degree of exophthalmos, CAS, spherical equivalent, axial length, blood free T4, T3, thyroid-stimulating hormone(TSH), and thyroid-stimulating immunoglobulin(TSI) concentrations, and choroidal thickness. To assess the disease activity, CAS was calculated by adding 1 point each for the following items; spontaneous eye pain, pain during eye movement, eyelid swelling, eyelid erythema, conjunctival injection, chemosis, and swollen caruncle [12].

**Fig. 1:**
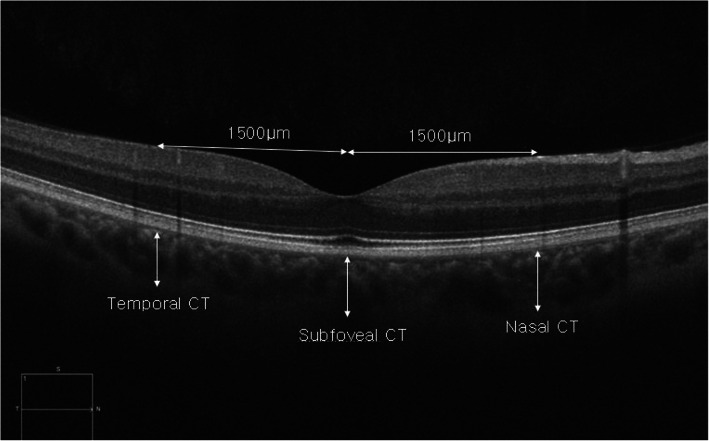
Measurements of choroidal thickness. Choroidal thickness was measured using images acquired in enhanced depth imaging (EDI) mode by cirrus HD-OCT (Carl Zeiss Meditec Inc., Dublin, CA, UAS). The vertical distance between the outermost border of the retinal pigment epithelium appears as a high-reflection line in OCT images and the inner border of the sclera was measured at the central fovea and points 1.5 mm nasal and 1.5 mm temporal from the central fovea using a caliper tool provided by OCT software

Choroidal thickness was measured using images acquired in enhanced depth imaging (EDI) mode by cirrus HD-OCT (Carl Zeiss Meditec Inc., Dublin, CA, UAS). For choroidal thickness, the vertical distance between the outermost border of retinal pigment epithelium appearing as high-reflection line in OCT images and inner border of the sclera was measured at the central fovea and points 1.5 mm nasal and 1.5 mm temporal from the central fovea using a caliper tool provided by OCT software (Fig. 1).

SPSS version 20.0 (IBM corp., Armonk, NY, USA) was used for statistical analysis and the measured values from the right eye of TED and control groups were used in the statistical analysis. The choroidal thickness between the two groups was compared by independent sample t-test with a statistical significance level of < 0.05. In addition, for variables with a P value of ≤ 0.15 in univariate regression analysis, multivariate regression analysis was performed to examine the factors that affect choroidal thickness.

## Results

The present study included 49 eyes each from the TED and control groups. The mean age of the target patients was 51.1 ± 13.2 and 50.2 ± 14.4 years in the TED and control groups, respectively, showing no significant difference between the two groups (*P* = 0.656). The mean spherical equivalent was − 1.3 ± 2.4 D and − 0.8 ± 1.7 D in the TED and control groups, respectively, showing no significant difference between the two groups (*P* = 0.112) (Table 1). The mean CAS was 1.4 ± 1.3 in the TED group with 81.6%, 8.2%, and 10.2% showing hyperthyroidism, hypothyroidism, and normal thyroid function, respectively (Table 2).

**Table 1:** Characteristics of thyroid eye disease (TED) group and Control group

	TED group (*n*=49)	Control group (*n*=49)	*P*-value
**Age (years)**	51.1±13.2	50.2±14.4	0.656
**Sex, n (%)**			
Male	15 (30.6%)	15 (30.6%)	
Female	34 (69.4%)	34 (69.4%)	
**SE (D)**	-1.3±2.4	-0.8±1.7	0.112
**AXL (mm)**	23.6±0.9	23.3±0.6	0.104

*TED* Thyroid eye disease; *SE *Spherical equivalent; *AXL* Axial length

**Table 2:** Characteristics of thyroid eye disease (TED) group

	TED group (*n*=49)
**Proptosis (mm)**	17.6±2.3
**CAS**	1.4±1.3
**IOP (mmHg)**	15.2±3.8
**Thyroid function status**	
Hyperthyroidism	40 (81.6%)
Hypothyroidism	4 (8.2%)
Euthyroidism	5 (10.2%)
**TFT**	
TSH (uIU/mL)	3.4±9.8
Free T4 (ng/dL)	1.5±0.5
T3 (ng/dL)	13.4±54.4
TSI (IU/L)	11.5±20.1

*TED* Thyroid eye disease; *CAS *Clinical activity score; *IOP* Intraocular pressure; *TFT* Thyroid function test; *TSH* Thyroid stimulating hormone; *TSI* Thyroid stimulating immunoglobulin

The mean central foveal choroidal thickness was 294.2 ± 71.4 µm and 261.1 ± 47.4 µm in the TED and control groups, respectively, while the mean temporal choroidal thickness was 267.6 ± 67.5 µm and 235.7 ± 41.3 µm in the TED and control groups, respectively, showing significant differences between the two groups (*P* = 0.011 and *P* = 0.008, respectively). Meanwhile, the mean nasal choroidal thickness was 232.1 ± 71.7 µm and 221.1 ± 59.9 µm in the TED and control groups, respectively, showing no significant difference between the two groups (*P* = 0.421)(Table 3).

**Table 3:** Choroidal thickness of thyroid eye disease (TED) group and Control group

	TED group (*n*=49)	Control group (*n*=49)	*P*-value
**Choroidal thickness**			
Subfoveal CT (μm)	294.2±71.4	261.1±47.4	0.011
Temporal CT (μm)	267.6±67.5	235.7±41.3	0.008
Nasal CT (μm)	232.1±71.7	221.1±59.9	0.421

*TED* Thyroid eye disease; *CT* Choroidal thickness

In the TED group, factors affecting central foveal choroidal thickness were analyzed using univariate regression analysis, and variables *P* value of ≤ 0.15, including age, axial length, degree of exophthalmos, and CAS were included in the multivariate regression analysis (Table 4). The multivariate simple regression analysis results showed that age (regression coefficient = -0.297, *P* = 0.036), axial length (regression coefficient = -0.330, *P* = 0.027), and exophthalmos (regression coefficient = 0.306, *P* = 0.040) were significant factors, whereas CAS (regression coefficient = -0.273, *P* = 0.059) was not a significant factor (Table 5).

**Table 4:** Univariate regression analysis of the association between subfoveal choroidal thickness and key variables

Variables	Univariate linear regression		
	Regression coefficient	R2	*P* value
**Age (years)**	-0.316	0.100	0.036
**SE (D)**	0.039	0.002	0.801
**AXL (mm)**	-0.246	0.060	0.108
**IOP (mmHg)**	-0.213	0.045	0.165
**Proptosis (mm)**	0.221	0.049	0.150
**CAS**	-0.245	0.060	0.110
**TSH (uIU/mL)**	-0.033	0.001	0.830
**Free T4 (ng/dL)**	0.003	0.000	0.986
**T3 (ng/dL)**	0.009	0.000	0.954
**TSI (IU/L)**	-0.155	0.024	0.316

*SE* Spherical equivalent; *AXL* Axial length; *IOP* Intraocular pressure; *CAS* Clinical activity score; *TSH* Thyroid stimulating hormone; *TSI* Thyroid stimulating immunoglobulin

**Table 5:** Multivariate regression analysis of the association between subfoveal choroidal thickness and key variables

Variables	Multivariate linear regression	
	Regression coefficient	*P* value
**Age (years)**	-0.297	0.036
**AXL (mm)**	-0.330	0.027
**Proptosis (mm)**	0.306	0.040
**CAS**	-0.273	0.059

*AXL* Axial length; *CAS *Clinical activity score

The factors affecting temporal choroidal thickness, including age, exophthalmos, and CAS, showed *P* values of ≤ 0.15 in univariate simple regression analysis, and were included in multivariate regression analysis (Table 6). The multivariate regression analysis results showed that age (Regression coefficient = -0.325, *P* = 0.029) and exophthalmos (Regression coefficient = 0.321, *P* = 0.032) were significant factors, whereas CAS (Regression coefficient = -0.204, *P* = 0.196) was not a significant factor (Table 7).

**Table 6:** Univariate regression analysis of the association between temporal choroidal thickness and key variables

Variables	Univariate linear regression		
	Regression coefficient	R2	*P* value
**Age (years)**	-0.302	0.079	0.046
**SE (D)**	0.065	0.004	0.677
**AXL (mm)**	-0.217	0.047	0.157
**IOP (mmHg)**	-0.140	0.020	0.363
**Proptosis (mm)**	0.254	0.064	0.097
**CAS**	-0.281	0.091	0.065
**TSH (uIU/mL)**	0.045	0.002	0.773
**Free T4 (ng/dL)**	-0.075	0.006	0.627
**T3 (ng/dL)**	-0.076	0.006	0.623
**TSI (IU/L)**	-0.143	0.021	0.354

*SE* Spherical equivalent; *AXL* Axial length; *IOP* Intraocular pressure; *CAS* Clinical activity score; *TSH* Thyroid stimulating hormone; *TSI* Thyroid stimulating immunoglobulin

**Table 7:** Multivariate regression analysis of the association between temporal choroidal thickness and key variables

Variables	Multivariate linear regression	
	Regression coefficient	*P* value
**Age (years)**	-0.325	0.029
**Proptosis (mm)**	0.321	0.032
**CAS**	-0.204	0.196

*CAS* Clinical activity score

In the univariate regression analysis on nasal choroidal thickness, there were no factors that showed significant association (Table 8).

**Table 8:** Univariate regression analysis of the association between nasal choroidal thickness and key variables

Variables	Univariate linear regression		
	Regression coefficient	R2	*P* value
**Age (years)**	-0.125	0.016	0.418
**SE (D)**	0.193	0.037	0.211
**AXL (mm)**	-0.015	0.000	0.922
**IOP (mmHg)**	-0.142	0.021	0.355
**Proptosis (mm)**	0.163	0.026	0.291
**CAS**	-0.128	0.016	0.408
**TSH (uIU/mL)**	0.032	0.001	0.838
**Free T4 (ng/dL)**	0.007	0.000	0.965
**T3 (ng/dL)**	-0.254	0.064	0.160
**TSI (IU/L)**	-0.131	0.017	0.395

*SE* Spherical equivalent, *AXL* Axial length, *IOP* Intraocular pressure, *CAS* Clinical activity score, *TSH* Thyroid stimulating hormone, *TSI* Thyroid stimulating immunoglobulin

## Discussion

Measurement of choroidal thickness has recently been made possible through imaging by OCT in EDI mode and various studies have reported on changes in choroidal thickness in various diseases. Fong et al. [5] and da Silva et al. [6] reported that choroidal thickness was thicker in patients with Vogt-Koyanagi-Harada disease, while Coskun et al. [7] reported that choroidal thickness was thicker in patients with Behcet disease. The studies reported that changes in choroidal thickness occurred in orbital inflammatory diseases due to choroidal infiltration of inflammatory cells, increased exudate, increased vascular leakage, and changes in orbital blood flow.

In patients with TED, orbital fibroblasts overexpressing thyroid-stimulating hormone receptors and insulin-like growth factor-1 receptors play a major role in orbital inflammation, production of extracellular matrix, and differentiation of adipose cells and myofibroblasts. Inflammatory cells usually infiltrate orbital fat tissue and extraocular muscles and cause orbital interstitial edema and enlargement of extraocular muscles due to the effects of inflammatory mediator cytokines. Nakase et al. [13] reported that patients with TED showed decreased orbital venous outflow, and it has been reported that the cause of decreased orbital venous outflow was due to increase in retrobulbar pressure to a level higher than normal venous pressure [14, 15]. It is believed that compression exerted by blood flow in the limited orbital space due to decrease in orbital venous outflow might be associated with an increase in choroidal thickness.

Another mechanism that may cause a change in choroidal thickness in TED cases is direct compression of the eyeball by enlarged extraocular muscles and orbital tissues. Choroidal folds may develop from external compression on the eyeball relayed to the choroid [16]. Jorge et al. [17] reported improvement in choroidal folds and recovery of visual acuity after orbital decompression in patients with TED, even if enlargement of extraocular muscles remained. Odrobina et al. [18] reported thickening of the central foveal choroidal thickness in long-term follow-up after scleral buckling procedure. The authors claimed that scleral buckling can cause a decrease in blood flow and stagnation of blood flow in the choroidal circulation, leading to increased choroidal pressure and central foveal choroidal thickness. In other words, choroidal folds may develop in patients with TED due to the pressure exerted by enlarged extraocular muscles and orbital tissues, and such direct pressure on the eyeball can cause increased choroidal thickness from stagnation of blood flow in the choroidal circulation, similar to the effect of scleral buckling.

In addition to age and axis length, the degree of exophthalmos showed significant association as a factor affecting central foveal choroidal thickness in the present study. A significant association was found even after adjusting for age and axis length. It is believed that these findings support the proposed mechanisms responsible for increased choroidal thickness in patients with TED, which were (1) increased retrobulbar pressure due to enlargement of orbital fat and extraocular muscles interference with orbital venous outflow and (2) mechanical pressure applied directly to the eyeball.

Some studies have reported on age-related changes in central foveal choroidal thickness. Ooto et al. [19], Margolis et al. [20], and Ding et al. [21] reported that an age-related decrease in choroidal thickness occurs in normal eyes and Ooto et al. [19] also reported that choroidal thickness decreases when axis length is increased. The present study found that an increase in age and axis length results in decreased central foveal choroidal thickness in patients with TED, which was consistent with previous studies of healthy subjects.

CAS is a useful tool for assessing the activity of TED. Caliskan et al. [2] reported that higher CAS was associated with thicker central foveal choroidal thickness. However, regression analyses in the present study did not show a significant association between CAS and choroidal thickness. Our findings were inconsistent with previously reported study results, which may be affected by different patient populations and not include patients with high CAS in the present study.

The present study was a cross-sectional study with analyses based on data from the initial TED diagnosis. OCT scans and thyroid function tests were performed at various time points to assess TED activity level and thyroid function, respectively. Therefore, we included patients with different thyroid function statuses and treatment states, which may have resulted in no significant correlation observed with blood TSH, free T4, T3, or TSI concentration and choroidal thickness. In this study, 40 patients with hyperthyroid state, 4 patients with hypothyroid state, and 5 patients with euthyroid state were included. Because of the difference in the number of patients between each group, comparisons between groups based on thyroid function status could not be made significantly. Moreover, additional studies are needed to understand the pathophysiology of why only central foveal and temporal choroidal thickness increased in the TED group. Through the study of the correlation between medial and lateral rectus muscle thickness and choroidal thickness, it will be helpful to understand some part of the pathophysiology of choroidal thickness increase in TED patients.

## Conclusions

In conclusion, our present study confirms that the central foveal and temporal choroidal thickness are significantly thicker in patients with inactive TED than in control subjects, while age, axial length, and degree of exophthalmos are major factors affecting choroidal thickness. It is believed that additional studies are needed to assess the clinical significance and pathophysiology of increased choroidal thickness in patients with TED according to disease duration and thyroid function.

## Data Availability

The datasets obtained and/or analyzed during the current study are available from the corresponding author on reasonable request.

## Abbreviations

TED: Thyroid eye disease.
CAS: Clinical activity scores.
TSH: Thyroid-stimulating hormone.
TSI: Thyroid-stimulating immunoglobulin.
EDI: Enhanced depth imaging.
HD-OCT: High-definition optical coherence tomography.
